# Supplementary material for: Cauda Equina Syndrome Core Outcome Set (CESCOS): An international patient and healthcare professional consensus for research studies
Source: PLoS One. 2020 Jan 10;15(1):e0225907. doi: 10.1371/journal.pone.0225907 (PMC6953762; doi:10.1371/journal.pone.0225907)
Supplement: S1 File — (DOCX) [file pone.0225907.s001.docx]

**TOPIC GUIDE CES QUALITATIVE INTERVIEWS**

**Aims and Objectives**

-To explore the patient experience of living with Cauda Equina Syndrome (CES)

-To ascertain what the patient feels are the most important outcomes that they are experiencing

-To ascertain what outcomes the patient feels are the most important to research in to improve CES management and aftercare

-To determine who should be key stakeholders

-Identify appropriate language to use for patient Delphi iterative process.

**Introduction (5-10 mins)**

Interviewer Name

Interviewer Occupation

Explain basic definition of CES

Explain looking for challenges experienced after the operation for CES

Explain expected intention, sensitive subjects and duration of interview and confidentiality

Confirm consent to qualitative interview

**Background (<5 mins)**

Interviewee name

Interviewee age

Interviewee occupation

Other medical conditions

When was your operation for CES?

**Interview questions (30 mins)**

How has your experience of this condition; Cauda Equina Syndrome been?

- What was it like before the back operation?

- What was it like after the back operation?

How do you feel your condition has been managed in hospital and in the community?

What were your expectations of life health-wise after the operation and what is the reality like?

Due to this condition what do you feel are the challenges to your health and wellbeing?

-bowel/bladder

-sex life

-back/ leg pain

-psychological

-**anxiety/fear**

-other

Would you be able to prioritise the importance of these for you now?

Was the importance of these different at earlier stages of the condition? (More relevant to those in the long term CES category)

Through this process of living with CES who else do you think has a good handle on the condition? If anyone? -Gauge other potential key stakeholders

Tell me a bit about the support you had for the condition?

**Closing remarks (5 mins)**

Considering your hospital, post op and follow up experience what would you have liked to change?

-support services

-more streamlined service with dedicated clinics

-research into timing for CES operations

-follow up as to the effects of long term CES

Offer the opportunity for the participant to comment on their interview transcript after transcription.
