# Supplementary material for: Cauda Equina Syndrome Core Outcome Set (CESCOS): An international patient and healthcare professional consensus for research studies
Source: PLoS One. 2020 Jan 10;15(1):e0225907. doi: 10.1371/journal.pone.0225907 (PMC6953762; doi:10.1371/journal.pone.0225907)
Supplement: S1 Table — (DOCX) [file pone.0225907.s002.docx]

**Patient and professional bodies contacted for Delphi recruitment**

| **Patient organisation** | **Healthcare professional organisation** |
| --- | --- |
| Cauda Equina Syndrome Association (CESA) | Society of British Neurological Surgeons |
| Cauda Equina Syndrome Foundation | Eurospine |
| Spinal Injuries Association | Canadian Spine society |
| Brain and Spine Foundation | International Spinal Cord Society |
|  | Spine Society of Australia |
|  | World federation of neuro-rehabilitation |
|  | British Society of rehabilitation medicine |
