# Supplementary material for: Cauda Equina Syndrome Core Outcome Set (CESCOS): An international patient and healthcare professional consensus for research studies
Source: PLoS One. 2020 Jan 10;15(1):e0225907. doi: 10.1371/journal.pone.0225907 (PMC6953762; doi:10.1371/journal.pone.0225907)
Supplement: S2 Table — (DOCX) [file pone.0225907.s003.docx]

**CESCOS (Cauda Equina Syndrome Core Outcome Set) list of outcomes for the Delphi Survey**

| **CLINICAL OUTCOMES** | | |
| --- | --- | --- |
| **Bladder Function (Nervous System)** | | |
| **Outcome** | **Plain Language description** | **Clinical description** |
| 1. Urinary retention | The patient cannot completely empty their bladder | Inability to completely empty bladder contents of urine |
| 1. Sensation of bladder fullness | The ability to sense that the bladder is full, which may be reduced in CES |  |
| 1. Incontinence of Urine | The patient has reduced control over when they urinate and wet themselves |  |
| 1. Urinary urgency | A sudden desire to pass urine |  |
| 1. Urinary frequency | The number of times the patient passes urine |  |
| **Bowel Function (Nervous System)** | | |
| **Outcome** | **Plain Language description** | **Clinical description** |
| 1. Constipation | Having difficulty passing stools | Difficulty passing faeces |
| 1. Faecal Incontinence | Less control over when a patient starts to pass stool causing “soiling” or “messing” oneself |  |
| 1. Abdominal distention | Tummy bloating |  |
| 1. Abdominal pain | Tummy pain |  |
| 1. Anal tone | A measure of the strength of the muscle in the back passage that prevents stool coming out. |  |
| **Sexual Function (Nervous System) and Life Impact (Physical Functioning)** | | |
| **Outcome** | **Plain Language description** | **Clinical description** |
| 1. Physical ability to have sexual intercourse | Physical problems with sexual intercourse such as difficulty achieving or maintaining an erection, numbness and reduced sensation in the genital region during sex or pain when having sex. | Erectile dysfunction, numbness or reduced genital sensation during sex and dyspareunia |
| **Muscle Strength (Nervous System)** | | |
| **Outcome** | **Plain Language description** | **Clinical description** |
| 1. Leg muscle strength | Reduction in the strength of the legs | Reduction in leg muscle power |
| 1. Foot drop | Weakness that prevents the patient lifting their foot off the floor | Weak muscles that dorsiflex at the ankle |
| 1. Reflexes | Automatic muscle reflexes usually checked in the legs during a medical exam by a doctor to see if they are present or not | Present or absent lower limb reflexes |
| **Sensory Loss (Nervous System)** | | |
| **Outcome** | **Plain Language description** | **Clinical description** |
| 1. Sensation in leg(s) | Reduced feeling or numbness in the leg(s) |  |
| 1. Sensation in genitals | Reduced feeling or numbness in the genitals |  |
| 1. Perineal sensation | Reduced feeling or numbness around the skin close to the anus | Reduced or loss of perineal sensation and saddle anaesthesia |
| **Pain (Nervous System)** | | |
| **Outcome** | **Plain Language description** | **Clinical description** |
| 1. Lower back pain | Pain in the lower back |  |
| 1. Pain in leg and/or feet | Pain in one or both legs (including “sciatica”) |  |
| **Muscle discomfort (Musculoskeletal)** | | |
| **Outcome** | **Plain Language description** | **Clinical description** |
| 1. Back stiffness | Feeling back is ‘stiff’, ‘tight’ or having uncomfortable muscle contractions |  |
| 1. Leg stiffness | Feeling legs are ‘stiff’, ‘tight’ or having uncomfortable muscle contractions |  |
| **General Outcomes** | | |
| **Outcome** | **Plain Language description** | **Clinical description** |
| 1. Fatigue | Feeling tired or energy levels are “low” |  |
| 1. Non-specific pain | Pain that is not limited to just one part of the body (such as back or legs) but is instead all over the body |  |

| **LIFE IMPACT** | | |
| --- | --- | --- |
| **Outcome** | **Plain Language description** | **Clinical description** |
| 1. Global Quality of Life | An overall measure how a person’s health effects their general wellbeing |  |
| 1. Occupation/ Role functioning | Impact of CES on the patient’s job or working life |  |
| 1. Social functioning | Impact of CES on relationships with partner, family and friends including ability to join in with social activities |  |
| 1. Ability to do Daily activities (Physical functioning) | Ability to do daily activities such as shopping, hoovering, ironing, laundry, driving become more difficult to do |  |
| 1. Mobility and Walking (Physical functioning) | Decreased ability to move around. Patients may require walking aids e.g. stick, Zimmer frame, wheelchair |  |
| 1. Difficulty with body posture (Physical functioning) | Difficulty with bending, lifting, standing and sitting, lying flat (difficulty sleeping). Here the difficulty to stand may lead to falls |  |
| 1. Sexual desire (Emotional functioning) | A reduced desire for sexual activity |  |
| 1. Anxiety (Emotional functioning) | Feeling of unease, worry or fear |  |
| 1. Isolation (Emotional functioning) | Feeling of loneliness, not “in touch” with society |  |
| 1. Low Mood and Depression (Emotional functioning) | Feeling “low” or feeling “blue”. This may include having suicidal ideas/ thoughts |  |

| **RESOURCES USE** | | |
| --- | --- | --- |
| **Outcome** | **Plain Language description** | **Clinical description** |
| 1. Hospital resources | Length and total cost of the hospital stay for the patient including investigations, medication, , surgery , staff time and other medical resources. |  |
| 1. Need for further intervention | The patient needs a repeat or further operation to help resolve CES or complications. |  |

| **DEATH** | |  |
| --- | --- | --- |
| **Outcome** | **Plain Language description** | **Clinical description** |
| 1. Death | Death within 30 days of an operation for CES either in hospital or after discharge |  |

| **ADVERSE EVENTS** | | |
| --- | --- | --- |
| **Outcome** | **Plain Language description** | **Clinical description** |
| 1. Complications | This would include any complication related to the operation or hospital stay excluding death. For example; wound infection, pressure sores, clots in the veins of the legs and/ or lungs, heart attack, blood transfusion, chest infection and recurrence of a spinal tumour |  |
